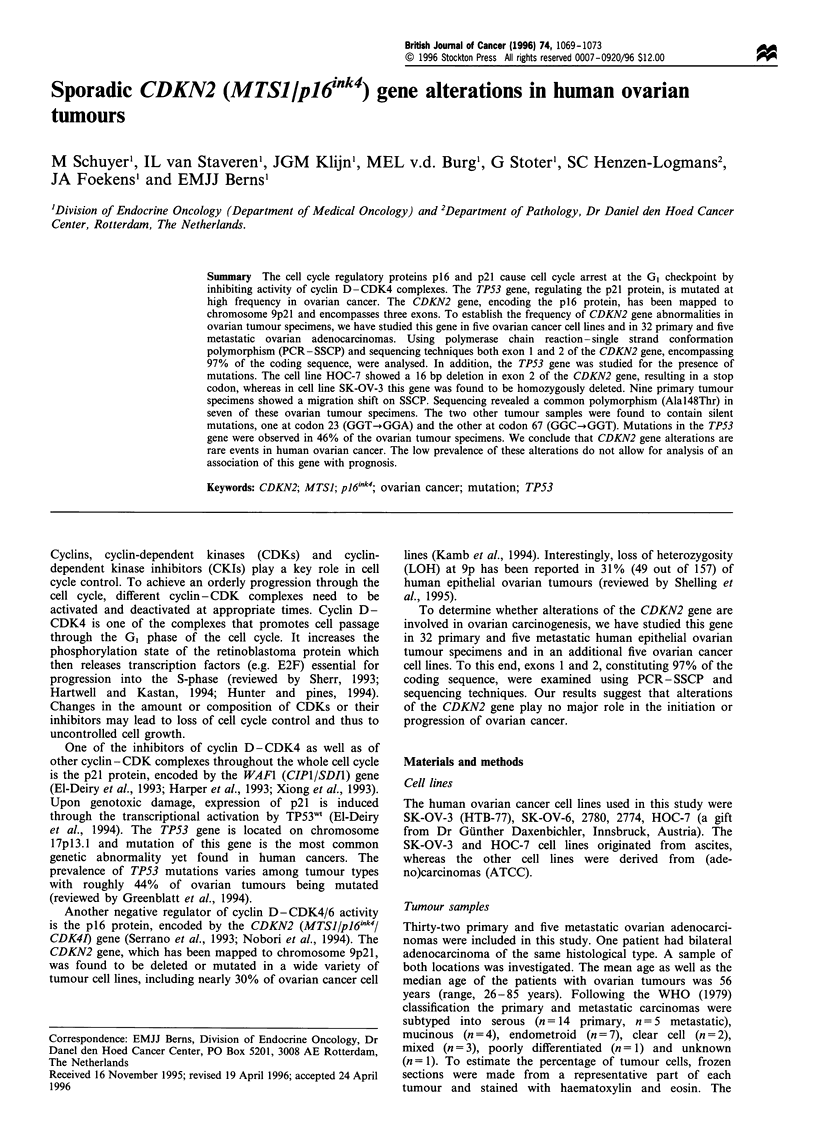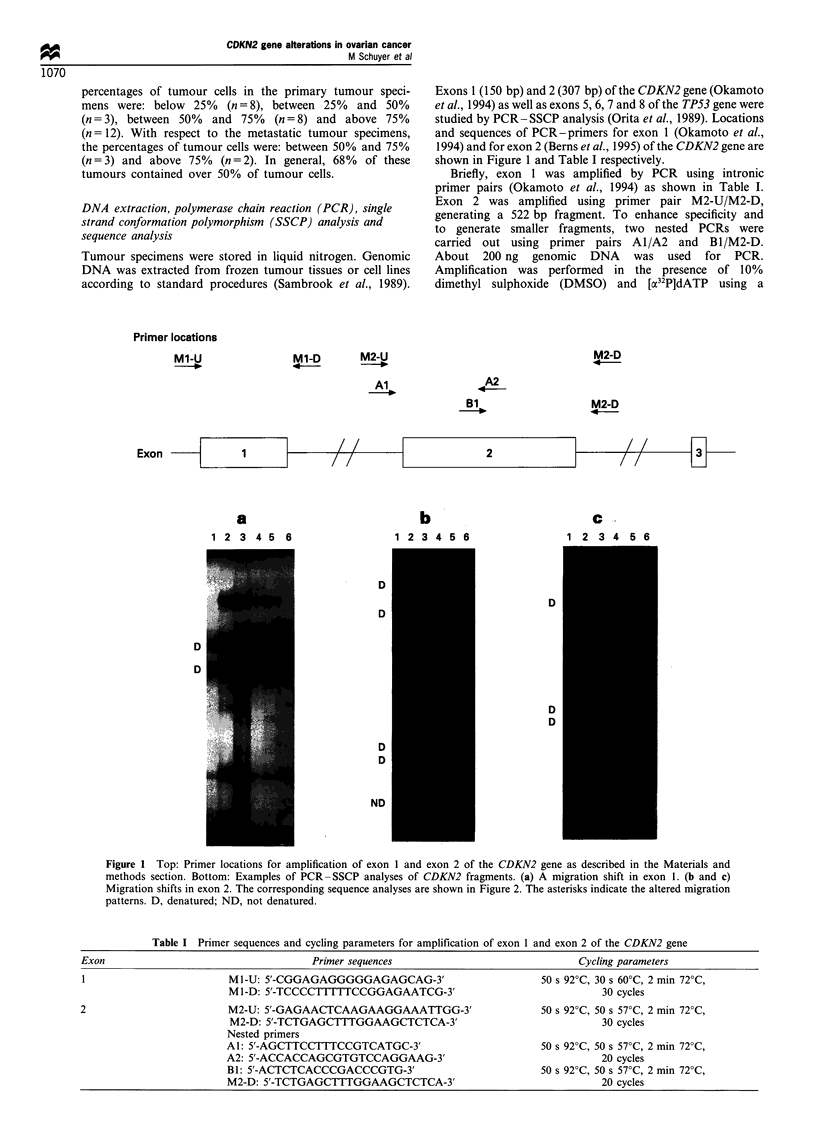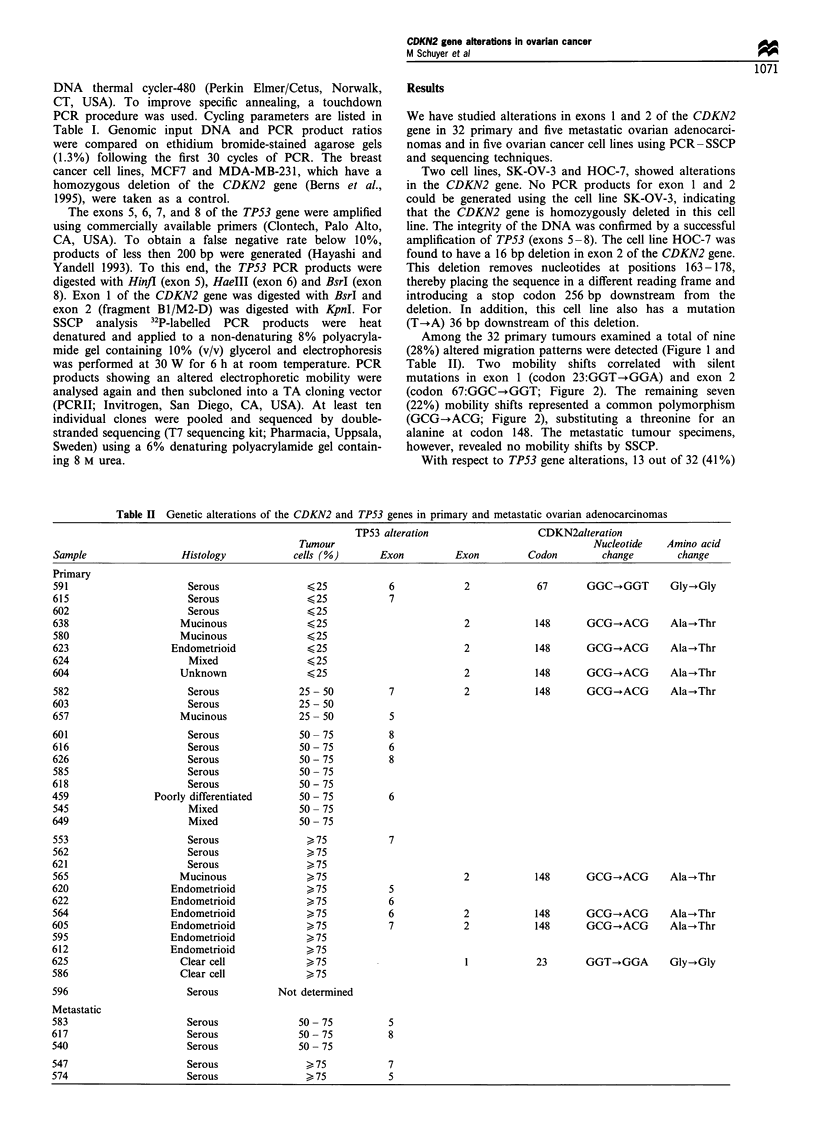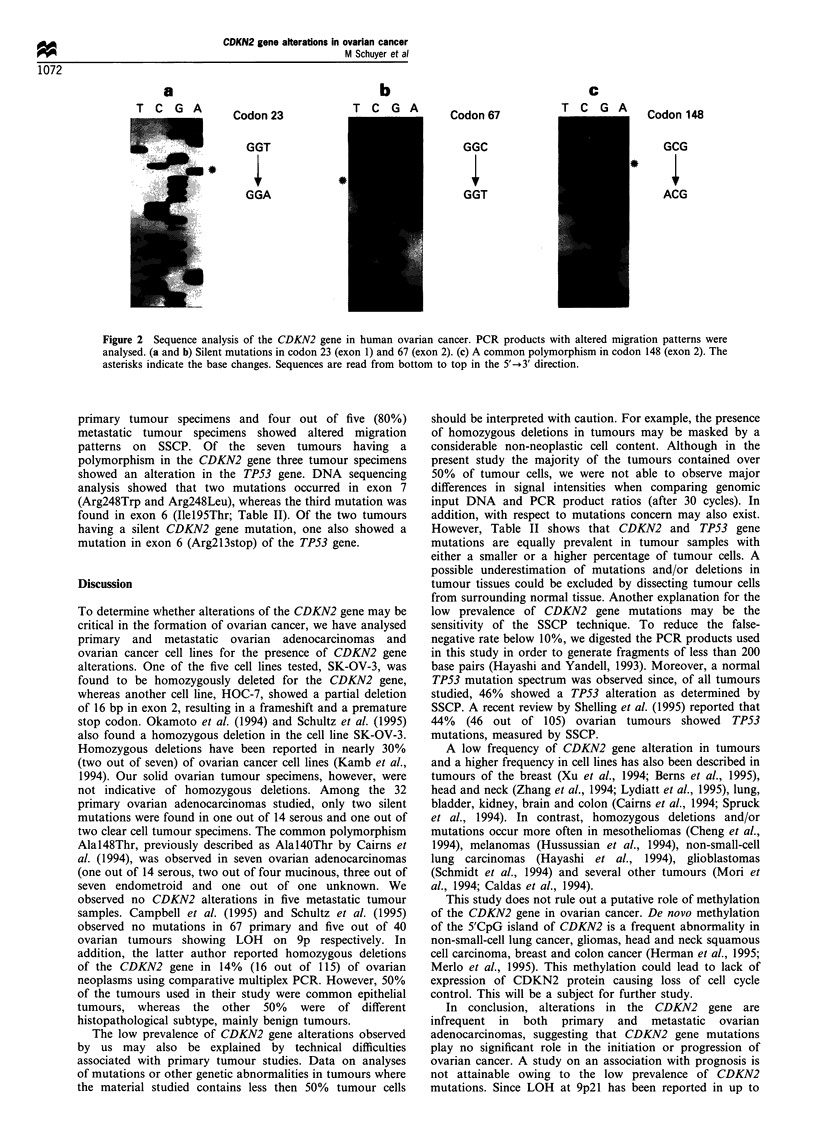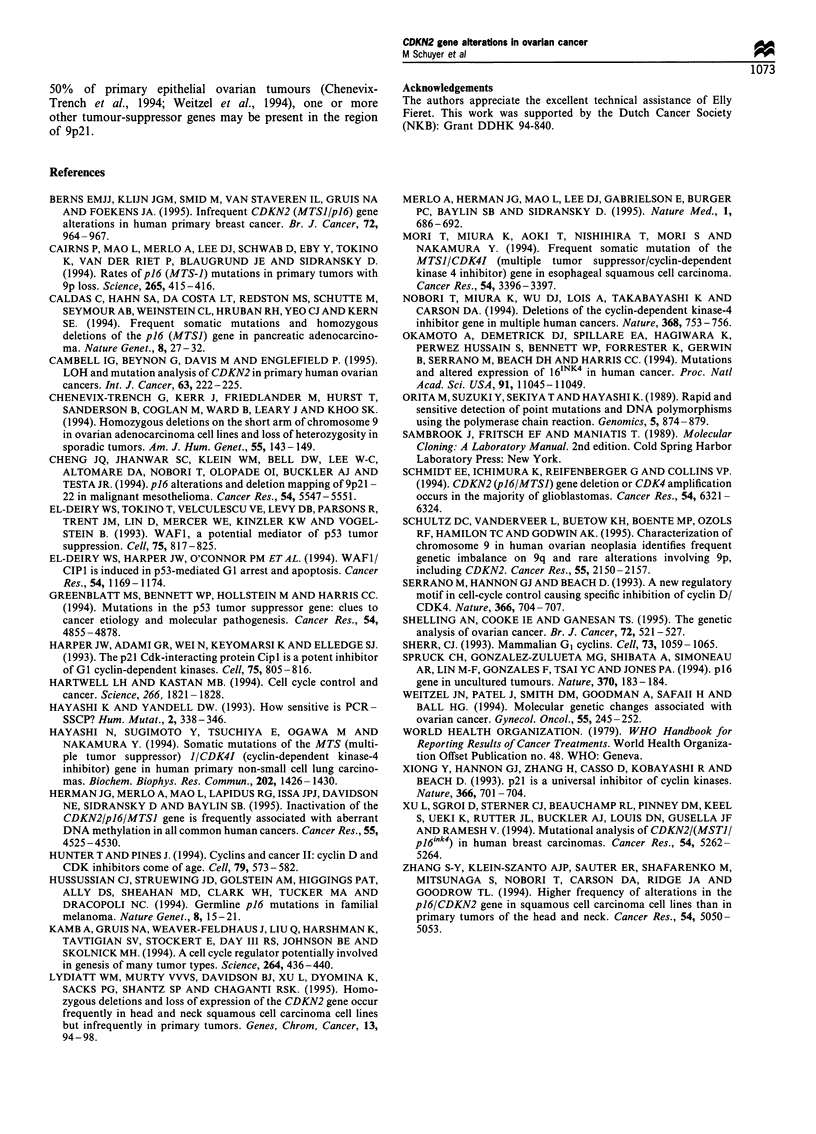# Sporadic CDKN2 (MTS1/p16ink4) gene alterations in human ovarian tumours.

**DOI:** 10.1038/bjc.1996.491

**Published:** 1996-10

**Authors:** M. Schuyer, I. L. van Staveren, J. G. Klijn, M. E. vd Burg, G. Stoter, S. C. Henzen-Logmans, J. A. Foekens, E. M. Berns

**Affiliations:** Division of Endocrine Oncology (Department of Medical Oncology), Dr Daniel den Hoed Cancer Center, Rotterdam, The Netherlands.

## Abstract

**Images:**


British Journal of Cancer (1996) 74, 1069-1073
© 1996 Stockton Press All rights reserved 0007-0920/96 $12.00

### Sporadic *CDKN2* (*MTS1/p16$^{ink4}$*) gene alterations in human ovarian tumours

M Schuyer[1], IL van Staveren[1], JGM Klijn[1], MEL v.d. Burg[1], G Stoter[1], SC Henzen-Logmans[2], JA Foekens[1] and EMJJ Berns[1]

[1]*Division of Endocrine Oncology (Department of Medical Oncology) and* [2]*Department of Pathology, Dr Daniel den Hoed Cancer Center, Rotterdam, The Netherlands.*

**Summary** The cell cycle regulatory proteins p16 and p21 cause cell cycle arrest at the $G_1$ checkpoint by inhibiting activity of cyclin D-CDK4 complexes. The *TP53* gene, regulating the p21 protein, is mutated at high frequency in ovarian cancer. The *CDKN2* gene, encoding the p16 protein, has been mapped to chromosome 9p21 and encompasses three exons. To establish the frequency of *CDKN2* gene abnormalities in ovarian tumour specimens, we have studied this gene in five ovarian cancer cell lines and in 32 primary and five metastatic ovarian adenocarcinomas. Using polymerase chain reaction-single strand conformation polymorphism (PCR-SSCP) and sequencing techniques both exon 1 and 2 of the *CDKN2* gene, encompassing 97% of the coding sequence, were analysed. In addition, the *TP53* gene was studied for the presence of mutations. The cell line HOC-7 showed a 16 bp deletion in exon 2 of the *CDKN2* gene, resulting in a stop codon, whereas in cell line SK-OV-3 this gene was found to be homozygously deleted. Nine primary tumour specimens showed a migration shift on SSCP. Sequencing revealed a common polymorphism (Ala148Thr) in seven of these ovarian tumour specimens. The two other tumour samples were found to contain silent mutations, one at codon 23 (GGT→GGA) and the other at codon 67 (GGC→GGT). Mutations in the *TP53* gene were observed in 46% of the ovarian tumour specimens. We conclude that *CDKN2* gene alterations are rare events in human ovarian cancer. The low prevalence of these alterations do not allow for analysis of an association of this gene with prognosis.

**Keywords:** *CDKN2*; *MTS1*; *p16$^{ink4}$*; ovarian cancer; mutation; *TP53*

Cyclins, cyclin-dependent kinases (CDKs) and cyclin-dependent kinase inhibitors (CKIs) play a key role in cell cycle control. To achieve an orderly progression through the cell cycle, different cyclin-CDK complexes need to be activated and deactivated at appropriate times. Cyclin D-CDK4 is one of the complexes that promotes cell passage through the $G_1$ phase of the cell cycle. It increases the phosphorylation state of the retinoblastoma protein which then releases transcription factors (e.g. E2F) essential for progression into the S-phase (reviewed by Sherr, 1993; Hartwell and Kastan, 1994; Hunter and pines, 1994). Changes in the amount or composition of CDKs or their inhibitors may lead to loss of cell cycle control and thus to uncontrolled cell growth.

One of the inhibitors of cyclin D-CDK4 as well as of other cyclin-CDK complexes throughout the whole cell cycle is the p21 protein, encoded by the *WAF*1 (*CIP*1/*SDI*1) gene (El-Deiry *et al.*, 1993; Harper *et al.*, 1993; Xiong *et al.*, 1993). Upon genotoxic damage, expression of p21 is induced through the transcriptional activation by $TP53^{wt}$ (El-Deiry *et al.*, 1994). The *TP53* gene is located on chromosome 17p13.1 and mutation of this gene is the most common genetic abnormality yet found in human cancers. The prevalence of *TP53* mutations varies among tumour types with roughly 44% of ovarian tumours being mutated (reviewed by Greenblatt *et al.*, 1994).

Another negative regulator of cyclin D-CDK4/6 activity is the p16 protein, encoded by the *CDKN2* (*MTS1/p16$^{ink4}$/CDK4I*) gene (Serrano *et al.*, 1993; Nobori *et al.*, 1994). The *CDKN2* gene, which has been mapped to chromosome 9p21, was found to be deleted or mutated in a wide variety of tumour cell lines, including nearly 30% of ovarian cancer cell lines (Kamb *et al.*, 1994). Interestingly, loss of heterozygosity (LOH) at 9p has been reported in 31% (49 out of 157) of human epithelial ovarian tumours (reviewed by Shelling *et al.*, 1995).

To determine whether alterations of the *CDKN2* gene are involved in ovarian carcinogenesis, we have studied this gene in 32 primary and five metastatic human epithelial ovarian tumour specimens and in an additional five ovarian cancer cell lines. To this end, exons 1 and 2, constituting 97% of the coding sequence, were examined using PCR-SSCP and sequencing techniques. Our results suggest that alterations of the *CDKN2* gene play no major role in the initiation or progression of ovarian cancer.

### Materials and methods

### *Cell lines*

The human ovarian cancer cell lines used in this study were SK-OV-3 (HTB-77), SK-OV-6, 2780, 2774, HOC-7 (a gift from Dr Günther Daxenbichler, Innsbruck, Austria). The SK-OV-3 and HOC-7 cell lines originated from ascites, whereas the other cell lines were derived from (adeno)carcinomas (ATCC).

### *Tumour samples*

Thirty-two primary and five metastatic ovarian adenocarcinomas were included in this study. One patient had bilateral adenocarcinoma of the same histological type. A sample of both locations was investigated. The mean age as well as the median age of the patients with ovarian tumours was 56 years (range, 26-85 years). Following the WHO (1979) classification the primary and metastatic carcinomas were subtyped into serous ($n=14$ primary, $n=5$ metastatic), mucinous ($n=4$), endometroid ($n=7$), clear cell ($n=2$), mixed ($n=3$), poorly differentiated ($n=1$) and unknown ($n=1$). To estimate the percentage of tumour cells, frozen sections were made from a representative part of each tumour and stained with haematoxylin and eosin. The

Correspondence: EMJJ Berns, Division of Endocrine Oncology, Dr Danel den Hoed Cancer Center, PO Box 5201, 3008 AE Rotterdam, The Netherlands
Received 16 November 1995; revised 19 April 1996; accepted 24 April 1996

percentages of tumour cells in the primary tumour specimens were: below 25% ($n=8$), between 25% and 50% ($n=3$), between 50% and 75% ($n=8$) and above 75% ($n=12$). With respect to the metastatic tumour specimens, the percentages of tumour cells were: between 50% and 75% ($n=3$) and above 75% ($n=2$). In general, 68% of these tumours contained over 50% of tumour cells.

*DNA extraction, polymerase chain reaction (PCR), single strand conformation polymorphism (SSCP) analysis and sequence analysis*

Tumour specimens were stored in liquid nitrogen. Genomic DNA was extracted from frozen tumour tissues or cell lines according to standard procedures (Sambrook *et al.*, 1989). Exons 1 (150 bp) and 2 (307 bp) of the *CDKN2* gene (Okamoto *et al.*, 1994) as well as exons 5, 6, 7 and 8 of the *TP53* gene were studied by PCR–SSCP analysis (Orita *et al.*, 1989). Locations and sequences of PCR–primers for exon 1 (Okamoto *et al.*, 1994) and for exon 2 (Berns *et al.*, 1995) of the *CDKN2* gene are shown in Figure 1 and Table I respectively.

Briefly, exon 1 was amplified by PCR using intronic primer pairs (Okamoto *et al.*, 1994) as shown in Table I. Exon 2 was amplified using primer pair M2-U/M2-D, generating a 522 bp fragment. To enhance specificity and to generate smaller fragments, two nested PCRs were carried out using primer pairs A1/A2 and B1/M2-D. About 200 ng genomic DNA was used for PCR. Amplification was performed in the presence of 10% dimethyl sulphoxide (DMSO) and [$\alpha^{32}$P]dATP using a

**Figure 1** Top: Primer locations for amplification of exon 1 and exon 2 of the *CDKN2* gene as described in the Materials and methods section. Bottom: Examples of PCR–SSCP analyses of *CDKN2* fragments. (**a**) A migration shift in exon 1. (**b** and **c**) Migration shifts in exon 2. The corresponding sequence analyses are shown in Figure 2. The asterisks indicate the altered migration patterns. D, denatured; ND, not denatured.

**Table I** Primer sequences and cycling parameters for amplification of exon 1 and exon 2 of the *CDKN2* gene

| *Exon* | *Primer sequences* | *Cycling parameters* |
|---|---|---|
| 1 | M1-U: 5′-CGGAGAGGGGGAGAGCAG-3′<br>M1-D: 5′-TCCCCTTTTTCCGGAGAATCG-3′ | 50 s 92°C, 30 s 60°C, 2 min 72°C, 30 cycles |
| 2 | M2-U: 5′-GAGAACTCAAGAAGGAAATTGG-3′<br>M2-D: 5′-TCTGAGCTTTGGAAGCTCTCA-3′ | 50 s 92°C, 50 s 57°C, 2 min 72°C, 30 cycles |
| | Nested primers | |
| | A1: 5′-AGCTTCCTTTCCGTCATGC-3′<br>A2: 5′-ACCACCAGCGTGTCCAGGAAG-3′ | 50 s 92°C, 50 s 57°C, 2 min 72°C, 20 cycles |
| | B1: 5′-ACTCTCACCCGACCCGTG-3′<br>M2-D: 5′-TCTGAGCTTTGGAAGCTCTCA-3′ | 50 s 92°C, 50 s 57°C, 2 min 72°C, 20 cycles |

DNA thermal cycler-480 (Perkin Elmer/Cetus, Norwalk, CT, USA). To improve specific annealing, a touchdown PCR procedure was used. Cycling parameters are listed in Table I. Genomic input DNA and PCR product ratios were compared on ethidium bromide-stained agarose gels (1.3%) following the first 30 cycles of PCR. The breast cancer cell lines, MCF7 and MDA-MB-231, which have a homozygous deletion of the *CDKN2* gene (Berns *et al*., 1995), were taken as a control.

The exons 5, 6, 7, and 8 of the *TP53* gene were amplified using commercially available primers (Clontech, Palo Alto, CA, USA). To obtain a false negative rate below 10%, products of less then 200 bp were generated (Hayashi and Yandell 1993). To this end, the *TP53* PCR products were digested with *Hinf*I (exon 5), *Hae*III (exon 6) and *Bsr*I (exon 8). Exon 1 of the *CDKN2* gene was digested with *Bsr*I and exon 2 (fragment B1/M2-D) was digested with *Kpn*I. For SSCP analysis $^{32}$P-labelled PCR products were heat denatured and applied to a non-denaturing 8% polyacrylamide gel containing 10% (v/v) glycerol and electrophoresis was performed at 30 W for 6 h at room temperature. PCR products showing an altered electrophoretic mobility were analysed again and then subcloned into a TA cloning vector (PCRII; Invitrogen, San Diego, CA, USA). At least ten individual clones were pooled and sequenced by double-stranded sequencing (T7 sequencing kit; Pharmacia, Uppsala, Sweden) using a 6% denaturing polyacrylamide gel containing 8 M urea.

### Results

We have studied alterations in exons 1 and 2 of the *CDKN2* gene in 32 primary and five metastatic ovarian adenocarcinomas and in five ovarian cancer cell lines using PCR-SSCP and sequencing techniques.

Two cell lines, SK-OV-3 and HOC-7, showed alterations in the *CDKN2* gene. No PCR products for exon 1 and 2 could be generated using the cell line SK-OV-3, indicating that the *CDKN2* gene is homozygously deleted in this cell line. The integrity of the DNA was confirmed by a successful amplification of *TP53* (exons 5-8). The cell line HOC-7 was found to have a 16 bp deletion in exon 2 of the *CDKN2* gene. This deletion removes nucleotides at positions 163-178, thereby placing the sequence in a different reading frame and introducing a stop codon 256 bp downstream from the deletion. In addition, this cell line also has a mutation (T→A) 36 bp downstream of this deletion.

Among the 32 primary tumours examined a total of nine (28%) altered migration patterns were detected (Figure 1 and Table II). Two mobility shifts correlated with silent mutations in exon 1 (codon 23:GGT→GGA) and exon 2 (codon 67:GGC→GGT; Figure 2). The remaining seven (22%) mobility shifts represented a common polymorphism (GCG→ACG; Figure 2), substituting a threonine for an alanine at codon 148. The metastatic tumour specimens, however, revealed no mobility shifts by SSCP.

With respect to *TP53* gene alterations, 13 out of 32 (41%)

**Table II** Genetic alterations of the *CDKN2* and *TP53* genes in primary and metastatic ovarian adenocarcinomas

| | | TP53 *alteration* | | CDKN2*alteration* | | | |
|---|---|---|---|---|---|---|---|
| *Sample* | *Histology* | *Tumour cells (%)* | *Exon* | *Exon* | *Codon* | *Nucleotide change* | *Amino acid change* |
| Primary | | | | | | | |
| 591 | Serous | ⩽25 | 6 | 2 | 67 | GGC→GGT | Gly→Gly |
| 615 | Serous | ⩽25 | 7 | | | | |
| 602 | Serous | ⩽25 | | | | | |
| 638 | Mucinous | ⩽25 | | 2 | 148 | GCG→ACG | Ala→Thr |
| 580 | Mucinous | ⩽25 | | | | | |
| 623 | Endometrioid | ⩽25 | | 2 | 148 | GCG→ACG | Ala→Thr |
| 624 | Mixed | ⩽25 | | | | | |
| 604 | Unknown | ⩽25 | | 2 | 148 | GCG→ACG | Ala→Thr |
| 582 | Serous | 25 - 50 | 7 | 2 | 148 | GCG→ACG | Ala→Thr |
| 603 | Serous | 25 - 50 | | | | | |
| 657 | Mucinous | 25 - 50 | 5 | | | | |
| 601 | Serous | 50 - 75 | 8 | | | | |
| 616 | Serous | 50 - 75 | 6 | | | | |
| 626 | Serous | 50 - 75 | 8 | | | | |
| 585 | Serous | 50 - 75 | | | | | |
| 618 | Serous | 50 - 75 | | | | | |
| 459 | Poorly differentiated | 50 - 75 | 6 | | | | |
| 545 | Mixed | 50 - 75 | | | | | |
| 649 | Mixed | 50 - 75 | | | | | |
| 553 | Serous | ⩾75 | 7 | | | | |
| 562 | Serous | ⩾75 | | | | | |
| 621 | Serous | ⩾75 | | | | | |
| 565 | Mucinous | ⩾75 | | 2 | 148 | GCG→ACG | Ala→Thr |
| 620 | Endometrioid | ⩾75 | 5 | | | | |
| 622 | Endometrioid | ⩾75 | 6 | | | | |
| 564 | Endometrioid | ⩾75 | 6 | 2 | 148 | GCG→ACG | Ala→Thr |
| 605 | Endometrioid | ⩾75 | 7 | 2 | 148 | GCG→ACG | Ala→Thr |
| 595 | Endometrioid | ⩾75 | | | | | |
| 612 | Endometrioid | ⩾75 | | | | | |
| 625 | Clear cell | ⩾75 | | 1 | 23 | GGT→GGA | Gly→Gly |
| 586 | Clear cell | ⩾75 | | | | | |
| 596 | Serous | Not determined | | | | | |
| Metastatic | | | | | | | |
| 583 | Serous | 50 - 75 | 5 | | | | |
| 617 | Serous | 50 - 75 | 8 | | | | |
| 540 | Serous | 50 - 75 | | | | | |
| 547 | Serous | ⩾75 | 7 | | | | |
| 574 | Serous | ⩾75 | 5 | | | | |

**Figure 2** Sequence analysis of the *CDKN2* gene in human ovarian cancer. PCR products with altered migration patterns were analysed. (**a** and **b**) Silent mutations in codon 23 (exon 1) and 67 (exon 2). (**c**) A common polymorphism in codon 148 (exon 2). The asterisks indicate the base changes. Sequences are read from bottom to top in the 5′→3′ direction.

primary tumour specimens and four out of five (80%) metastatic tumour specimens showed altered migration patterns on SSCP. Of the seven tumours having a polymorphism in the *CDKN2* gene three tumour specimens showed an alteration in the *TP53* gene. DNA sequencing analysis showed that two mutations occurred in exon 7 (Arg248Trp and Arg248Leu), whereas the third mutation was found in exon 6 (Ile195Thr; Table II). Of the two tumours having a silent *CDKN2* gene mutation, one also showed a mutation in exon 6 (Arg213stop) of the *TP53* gene.

### Discussion

To determine whether alterations of the *CDKN2* gene may be critical in the formation of ovarian cancer, we have analysed primary and metastatic ovarian adenocarcinomas and ovarian cancer cell lines for the presence of *CDKN2* gene alterations. One of the five cell lines tested, SK-OV-3, was found to be homozygously deleted for the *CDKN2* gene, whereas another cell line, HOC-7, showed a partial deletion of 16 bp in exon 2, resulting in a frameshift and a premature stop codon. Okamoto *et al.* (1994) and Schultz *et al.* (1995) also found a homozygous deletion in the cell line SK-OV-3. Homozygous deletions have been reported in nearly 30% (two out of seven) of ovarian cancer cell lines (Kamb *et al.*, 1994). Our solid ovarian tumour specimens, however, were not indicative of homozygous deletions. Among the 32 primary ovarian adenocarcinomas studied, only two silent mutations were found in one out of 14 serous and one out of two clear cell tumour specimens. The common polymorphism Ala148Thr, previously described as Ala140Thr by Cairns *et al.* (1994), was observed in seven ovarian adenocarcinomas (one out of 14 serous, two out of four mucinous, three out of seven endometroid and one out of one unknown. We observed no *CDKN2* alterations in five metastatic tumour samples. Campbell *et al.* (1995) and Schultz *et al.* (1995) observed no mutations in 67 primary and five out of 40 ovarian tumours showing LOH on 9p respectively. In addition, the latter author reported homozygous deletions of the *CDKN2* gene in 14% (16 out of 115) of ovarian neoplasms using comparative multiplex PCR. However, 50% of the tumours used in their study were common epithelial tumours, whereas the other 50% were of different histopathological subtype, mainly benign tumours.

The low prevalence of *CDKN2* gene alterations observed by us may also be explained by technical difficulties associated with primary tumour studies. Data on analyses of mutations or other genetic abnormalities in tumours where the material studied contains less then 50% tumour cells should be interpreted with caution. For example, the presence of homozygous deletions in tumours may be masked by a considerable non-neoplastic cell content. Although in the present study the majority of the tumours contained over 50% of tumour cells, we were not able to observe major differences in signal intensities when comparing genomic input DNA and PCR product ratios (after 30 cycles). In addition, with respect to mutations concern may also exist. However, Table II shows that *CDKN2* and *TP53* gene mutations are equally prevalent in tumour samples with either a smaller or a higher percentage of tumour cells. A possible underestimation of mutations and/or deletions in tumour tissues could be excluded by dissecting tumour cells from surrounding normal tissue. Another explanation for the low prevalence of *CDKN2* gene mutations may be the sensitivity of the SSCP technique. To reduce the false-negative rate below 10%, we digested the PCR products used in this study in order to generate fragments of less than 200 base pairs (Hayashi and Yandell, 1993). Moreover, a normal *TP53* mutation spectrum was observed since, of all tumours studied, 46% showed a *TP53* alteration as determined by SSCP. A recent review by Shelling *et al.* (1995) reported that 44% (46 out of 105) ovarian tumours showed *TP53* mutations, measured by SSCP.

A low frequency of *CDKN2* gene alteration in tumours and a higher frequency in cell lines has also been described in tumours of the breast (Xu *et al.*, 1994; Berns *et al.*, 1995), head and neck (Zhang *et al.*, 1994; Lydiatt *et al.*, 1995), lung, bladder, kidney, brain and colon (Cairns *et al.*, 1994; Spruck *et al.*, 1994). In contrast, homozygous deletions and/or mutations occur more often in mesotheliomas (Cheng *et al.*, 1994), melanomas (Hussussian *et al.*, 1994), non-small-cell lung carcinomas (Hayashi *et al.*, 1994), glioblastomas (Schmidt *et al.*, 1994) and several other tumours (Mori *et al.*, 1994; Caldas *et al.*, 1994).

This study does not rule out a putative role of methylation of the *CDKN2* gene in ovarian cancer. *De novo* methylation of the 5′CpG island of *CDKN2* is a frequent abnormality in non-small-cell lung cancer, gliomas, head and neck squamous cell carcinoma, breast and colon cancer (Herman *et al.*, 1995; Merlo *et al.*, 1995). This methylation could lead to lack of expression of CDKN2 protein causing loss of cell cycle control. This will be a subject for further study.

In conclusion, alterations in the *CDKN2* gene are infrequent in both primary and metastatic ovarian adenocarcinomas, suggesting that *CDKN2* gene mutations play no significant role in the initiation or progression of ovarian cancer. A study on an association with prognosis is not attainable owing to the low prevalence of *CDKN2* mutations. Since LOH at 9p21 has been reported in up to

50% of primary epithelial ovarian tumours (Chenevix-Trench *et al.*, 1994; Weitzel *et al.*, 1994), one or more other tumour-suppressor genes may be present in the region of 9p21.

**Acknowledgements**
The authors appreciate the excellent technical assistance of Elly Fieret. This work was supported by the Dutch Cancer Society (NKB): Grant DDHK 94-840.

### References

BERNS EMJJ, KLIJN JGM, SMID M, VAN STAVEREN IL, GRUIS NA AND FOEKENS JA. (1995). Infrequent *CDKN2* (*MTS1/p16*) gene alterations in human primary breast cancer. *Br. J. Cancer*, **72**, 964–967.

CAIRNS P, MAO L, MERLO A, LEE DJ, SCHWAB D, EBY Y, TOKINO K, VAN DER RIET P, BLAUGRUND JE AND SIDRANSKY D. (1994). Rates of *p16* (*MTS-1*) mutations in primary tumors with 9p loss. *Science*, **265**, 415–416.

CALDAS C, HAHN SA, DA COSTA LT, REDSTON MS, SCHUTTE M, SEYMOUR AB, WEINSTEIN CL, HRUBAN RH, YEO CJ AND KERN SE. (1994). Frequent somatic mutations and homozygous deletions of the *p16* (*MTS1*) gene in pancreatic adenocarcinoma. *Nature Genet.*, **8**, 27–32.

CAMBELL IG, BEYNON G, DAVIS M AND ENGLEFIELD P. (1995). LOH and mutation analysis of *CDKN2* in primary human ovarian cancers. *Int. J. Cancer*, **63**, 222–225.

CHENEVIX-TRENCH G, KERR J, FRIEDLANDER M, HURST T, SANDERSON B, COGLAN M, WARD B, LEARY J AND KHOO SK. (1994). Homozygous deletions on the short arm of chromosome 9 in ovarian adenocarcinoma cell lines and loss of heterozygosity in sporadic tumors. *Am. J. Hum. Genet.*, **55**, 143–149.

CHENG JQ, JHANWAR SC, KLEIN WM, BELL DW, LEE W-C, ALTOMARE DA, NOBORI T, OLOPADE OI, BUCKLER AJ AND TESTA JR. (1994). *p16* alterations and deletion mapping of 9p21–22 in malignant mesothelioma. *Cancer Res.*, **54**, 5547–5551.

EL-DEIRY WS, TOKINO T, VELCULESCU VE, LEVY DB, PARSONS R, TRENT JM, LIN D, MERCER WE, KINZLER KW AND VOGELSTEIN B. (1993). WAF1, a potential mediator of p53 tumor suppression. *Cell*, **75**, 817–825.

EL-DEIRY WS, HARPER JW, O'CONNOR PM *ET AL.* (1994). WAF1/CIP1 is induced in p53-mediated G1 arrest and apoptosis. *Cancer Res.*, **54**, 1169–1174.

GREENBLATT MS, BENNETT WP, HOLLSTEIN M AND HARRIS CC. (1994). Mutations in the p53 tumor suppressor gene: clues to cancer etiology and molecular pathogenesis. *Cancer Res.*, **54**, 4855–4878.

HARPER JW, ADAMI GR, WEI N, KEYOMARSI K AND ELLEDGE SJ. (1993). The p21 Cdk-interacting protein Cip1 is a potent inhibitor of G1 cyclin-dependent kinases. *Cell*, **75**, 805–816.

HARTWELL LH AND KASTAN MB. (1994). Cell cycle control and cancer. *Science*, *266*, 1821–1828.

HAYASHI K AND YANDELL DW. (1993). How sensitive is PCR–SSCP? *Hum. Mutat.*, **2**, 338–346.

HAYASHI N, SUGIMOTO Y, TSUCHIYA E, OGAWA M AND NAKAMURA Y. (1994). Somatic mutations of the *MTS* (multiple tumor suppressor) *1/CDK4I* (cyclin-dependent kinase-4 inhibitor) gene in human primary non-small cell lung carcinomas. *Biochem. Biophys. Res. Commun.*, **202**, 1426–1430.

HERMAN JG, MERLO A, MAO L, LAPIDUS RG, ISSA JPJ, DAVIDSON NE, SIDRANSKY D AND BAYLIN SB. (1995). Inactivation of the *CDKN2/p16/MTS1* gene is frequently associated with aberrant DNA methylation in all common human cancers. *Cancer Res.*, **55**, 4525–4530.

HUNTER T AND PINES J. (1994). Cyclins and cancer II: cyclin D and CDK inhibitors come of age. *Cell*, **79**, 573–582.

HUSSUSSIAN CJ, STRUEWING JD, GOLSTEIN AM, HIGGINGS PAT, ALLY DS, SHEAHAN MD, CLARK WH, TUCKER MA AND DRACOPOLI NC. (1994). Germline *p16* mutations in familial melanoma. *Nature Genet.*, **8**, 15–21.

KAMB A, GRUIS NA, WEAVER-FELDHAUS J, LIU Q, HARSHMAN K, TAVTIGIAN SV, STOCKERT E, DAY III RS, JOHNSON BE AND SKOLNICK MH. (1994). A cell cycle regulator potentially involved in genesis of many tumor types. *Science*, **264**, 436–440.

LYDIATT WM, MURTY VVVS, DAVIDSON BJ, XU L, DYOMINA K, SACKS PG, SHANTZ SP AND CHAGANTI RSK. (1995). Homozygous deletions and loss of expression of the *CDKN2* gene occur frequently in head and neck squamous cell carcinoma cell lines but infrequently in primary tumors. *Genes, Chrom, Cancer*, **13**, 94–98.

MERLO A, HERMAN JG, MAO L, LEE DJ, GABRIELSON E, BURGER PC, BAYLIN SB AND SIDRANSKY D. (1995). *Nature Med.*, **1**, 686–692.

MORI T, MIURA K, AOKI T, NISHIHIRA T, MORI S AND NAKAMURA Y. (1994). Frequent somatic mutation of the *MTS1/CDK4I* (multiple tumor suppressor/cyclin-dependent kinase 4 inhibitor) gene in esophageal squamous cell carcinoma. *Cancer Res.*, **54**, 3396–3397.

NOBORI T, MIURA K, WU DJ, LOIS A, TAKABAYASHI K AND CARSON DA. (1994). Deletions of the cyclin-dependent kinase-4 inhibitor gene in multiple human cancers. *Nature*, **368**, 753–756.

OKAMOTO A, DEMETRICK DJ, SPILLARE EA, HAGIWARA K, PERWEZ HUSSAIN S, BENNETT WP, FORRESTER K, GERWIN B, SERRANO M, BEACH DH AND HARRIS CC. (1994). Mutations and altered expression of $16^{INK4}$ in human cancer. *Proc. Natl Acad. Sci. USA*, **91**, 11045–11049.

ORITA M, SUZUKI Y, SEKIYA T AND HAYASHI K. (1989). Rapid and sensitive detection of point mutations and DNA polymorphisms using the polymerase chain reaction. *Genomics*, **5**, 874–879.

SAMBROOK J, FRITSCH EF AND MANIATIS T. (1989). *Molecular Cloning: A Laboratory Manual.* 2nd edition. Cold Spring Harbor Laboratory Press: New York.

SCHMIDT EE, ICHIMURA K, REIFENBERGER G AND COLLINS VP. (1994). *CDKN2* (*p16/MTS1*) gene deletion or *CDK4* amplification occurs in the majority of glioblastomas. *Cancer Res.*, **54**, 6321–6324.

SCHULTZ DC, VANDERVEER L, BUETOW KH, BOENTE MP, OZOLS RF, HAMILON TC AND GODWIN AK. (1995). Characterization of chromosome 9 in human ovarian neoplasia identifies frequent genetic imbalance on 9q and rare alterations involving 9p, including *CDKN2*. *Cancer Res.*, **55**, 2150–2157.

SERRANO M, HANNON GJ AND BEACH D. (1993). A new regulatory motif in cell-cycle control causing specific inhibition of cyclin D/CDK4. *Nature*, **366**, 704–707.

SHELLING AN, COOKE IE AND GANESAN TS. (1995). The genetic analysis of ovarian cancer. *Br. J. Cancer*, **72**, 521–527.

SHERR, CJ. (1993). Mammalian $G_1$ cyclins. *Cell*, **73**, 1059–1065.

SPRUCK CH, GONZALEZ-ZULUETA MG, SHIBATA A, SIMONEAU AR, LIN M-F, GONZALES F, TSAI YC AND JONES PA. (1994). p16 gene in uncultured tumours. *Nature*, **370**, 183–184.

WEITZEL JN, PATEL J, SMITH DM, GOODMAN A, SAFAII H AND BALL HG. (1994). Molecular genetic changes associated with ovarian cancer. *Gynecol. Oncol.*, **55**, 245–252.

WORLD HEALTH ORGANIZATION. (1979). *WHO Handbook for Reporting Results of Cancer Treatments.* World Health Organization Offset Publication no. 48. WHO: Geneva.

XIONG Y, HANNON GJ, ZHANG H, CASSO D, KOBAYASHI R AND BEACH D. (1993). p21 is a universal inhibitor of cyclin kinases. *Nature*, **366**, 701–704.

XU L, SGROI D, STERNER CJ, BEAUCHAMP RL, PINNEY DM, KEEL S, UEKI K, RUTTER JL, BUCKLER AJ, LOUIS DN, GUSELLA JF AND RAMESH V. (1994). Mutational analysis of *CDKN2*/(*MST1*/$p16^{ink4}$) in human breast carcinomas. *Cancer Res.*, **54**, 5262–5264.

ZHANG S-Y, KLEIN-SZANTO AJP, SAUTER ER, SHAFARENKO M, MITSUNAGA S, NOBORI T, CARSON DA, RIDGE JA AND GOODROW TL. (1994). Higher frequency of alterations in the *p16/CDKN2* gene in squamous cell carcinoma cell lines than in primary tumors of the head and neck. *Cancer Res.*, **54**, 5050–5053.